# More than double the fun with two-photon excitation microscopy

**DOI:** 10.1038/s42003-024-06057-0

**Published:** 2024-03-26

**Authors:** Peter Luu, Scott E. Fraser, Falk Schneider

**Affiliations:** 1 Translational Imaging Center, Michelson Center for Convergent Bioscience, University of Southern California, Los Angeles, CA 90089 USA; 2 Department of Biological Sciences, Division of Molecular and Computational Biology, University of Southern California, Los Angeles, CA 90089 USA; 3 Alfred Mann Department of Biomedical Engineering, University of Southern California, Los Angeles, CA 90089 USA; 4 Dana and David Dornsife College of Letters, Arts and Sciences, University of Southern California, Los Angeles, CA 90089 USA

**Keywords:** Fluorescence imaging, Multiphoton microscopy

## Abstract

For generations researchers have been observing the dynamic processes of life through the lens of a microscope. This has offered tremendous insights into biological phenomena that span multiple orders of time- and length-scales ranging from the pure magic of molecular reorganization at the membrane of immune cells, to cell migration and differentiation during development or wound healing. Standard fluorescence microscopy techniques offer glimpses at such processes in vitro, however, when applied in intact systems, they are challenged by reduced signal strengths and signal-to-noise ratios that result from deeper imaging. As a remedy, two-photon excitation (TPE) microscopy takes a special place, because it allows us to investigate processes in vivo, in their natural environment, even in a living animal. Here, we review the fundamental principles underlying TPE aimed at basic and advanced microscopy users interested in adopting TPE for intravital imaging. We focus on applications in neurobiology, present current trends towards faster, wider and deeper imaging, discuss the combination with photon counting technologies for metabolic imaging and spectroscopy, as well as highlight outstanding issues and drawbacks in development and application of these methodologies.

## Abstract (web summary)

This review introduces the basic principle of fluorescence and two-photon excitation microscopy that is aimed at novice and advanced microscopists highlighting current techniques, trends, and practical guides for live imaging optimization.

## Introduction

Two-photon excitation (TPE) laser scanning microscopy (LSM) has evolved from a custom tool to a broadly available imaging modality in the life sciences. Number of users and applications have grown dramatically in the decades since it was demonstrated by Winfried Denk and his coworker James “Jim” H. Strickler in the Webb lab^1^. TPE microscopy has emerged as the gold standard for deep tissue and intravital imaging as well as for metabolic studies. Exemplary applications include imaging of cultured cells^2^, imaging of neuronal activity in single cells and tissue slices^3,4^ as well as model organisms such as mice^5^, rats^6^, or zebrafish^7,8^, and deep-tissue imaging^9^, even in challenging settings such as following immune cell trafficking in intact lymph nodes^10^. In this review, we will first cover the basics of fluorescence and TPE microscopy and then present many of the growing sets of applications in biological imaging along with cutting-edge technical developments.

## Principles of fluorescence and TPE microscopy

**Fig. 1:**
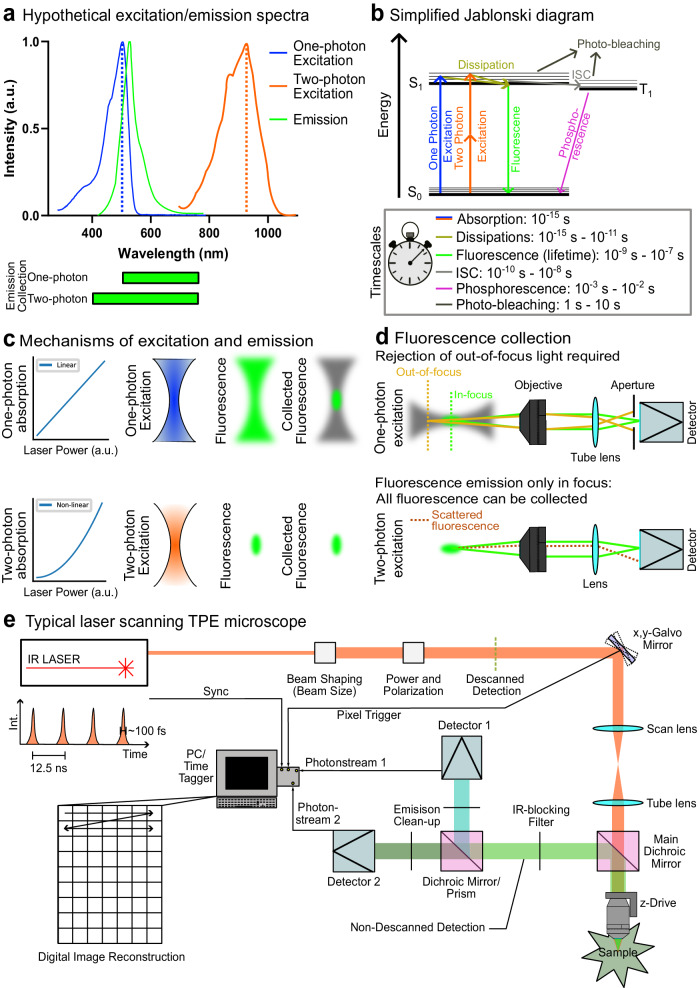
Introduction to TPE microscopy. **a** Hypothetical excitation (solid blue and orange lines) and emission spectra (solid green line) of a fluorophore in one-photon and two-photon excitation (one-photon and two-photon excitation maxima indicated as blue and orange dashed lines, respectively) and typical emission collection (green bars, bottom). **b** Simplified Jablonski diagram showing the ground state (S_0_), first excited state (S_1_), triplet state (T_1_) and vibrational states (thin lines). Absorption of one or two photons of the right energy excites the molecule and allows for fluorescence and phosphorescence (return to ground state) after energy dissipation through vibrational states. Inter system crossing (ICS) can take the molecule into a long-lived dark-state. From the excited states molecules can react further by photo-bleaching (loss of fluorescence). The bottom panel outlines approximate time-scales for the processes shown in the Jablonski diagram. **c** Principles of one-photon and two-photon excitation and emission at the focal plane and out-of-focus. One-photon absorption increases linearly with incident laser light whereas two-photon absorption increases non-linearly (quadratically) with incident laser light (left panels). In TPE microscopy, this allows for fluorescence excitation only at the focal spot. In one-photon excitation, this highlights the increased photo-bleaching due to out-of-focus excitation (gray). Further this explains the necessity for an aperture in standard confocal detection (**d**). **d** Scheme of a typical detection in one- and two-photon excitation experiments: in one-photon configuration, a pinhole rejects out-of-focus light whereas in TPE microscopy fluorescence only originates in the focal plane, thus additional scattered photons can be collected, increasing detection efficiency. **e** Scheme of a typical TPE laser scanning microscope. Depicted are beam path, TPE laser properties, non-descanned detection, and digital image reconstruction. For comparison with conventional confocal LSM, the position of the descanned detection (before the galvo mirror) is indicated by a green dashed line (meaning in descanned detection the main dichroic mirror and the detectors would be moved to this position).

Fluorescence microscopy provides molecular sensitivity and specificity to image a fluorescently labeled species against background. Typically, a fluorophore absorbs a single photon and emits a single photon of a longer wavelength, causing a red shift between excitation and emission termed the Stokes’ shift (Fig. 1a, b)^11^. This Stokes’ shift is the foundation for contrast in fluorescence microscopy as dichroic mirrors and appropriate filters can be used to separate the excitation and emission wavelengths. The time delay of a few nanoseconds (ns) between excitation and emission required for cycling a typical fluorophore from ground state to excited state and back permits Fluorescence Lifetime Imaging Microscopy (FLIM), which exploits this characteristic delay as an additional source of contrast (Fig. 1b). Other photophysical effects such as phosphorescence (as a result of intersystem-crossing, ICS) might be used to generate contrast but are less popular because they offer fewer photons per unit time^12^.

Two-photon excitation (TPE) microscopy is made possible by a fluorophore simultaneously absorbing two photons of about double the wavelength (half the energy) required for one-photon excitation (Fig. 1a, b). This was first predicted by Maria Goeppert-Mayer in the 1930s^13^, whose pioneering work is recognised by naming the unit of the probability of two photon absorption (TPA) after her, GM units (1 GM = 10^−^^50^ cm^4^⋅s)^14^. The first experimental demonstration of fluorescence from TPE was achieved by Kaiser and Garret in europium doped calcium fluoride crystals (CaF_2_:Eu^2+^) decades later^15^. Because of the need for two photons to excite the fluorophore, the probability of absorption (and subsequent fluorescence emission) depends on the square of the number of photons reaching the fluorophore simultaneously; thus, two-photon excitation exhibits a non-linear (quadratic) relationship to the excitation intensity unlike the linear relationship in one-photon excitation^14^ (Fig. 1c, left). The two-photon absorption cross-sections of typical fluorophores require large, instantaneous photon densities, which are usually achieved by tightly focusing the beam (mW power) of a short-pulsed laser (~100s fs pulse width, typically pulsing at a repetition rate of ~80 MHz), concentrating photons both spatially and temporally. Because the photon density falls off by the square of the distance from the focus, excitation (and fluorescence emission) falls off by the fourth power of the distance from the focus of the infra-red (IR) laser beam^16^. This provides optical sectioning comparable to a confocal (one-photon excitation) microscope which, however, requires a confocal aperture, pinhole, to reject fluorescence excited above and below the optical section in focus (Fig. 1c, d). Given the selective excitation of the TPE beam, the excited fluorescence can be collected far more efficiently because scattered emission can also be collected (Fig. 1d). Thus, TPE reduces out-of-focus excitation (reducing photobleaching and phototoxicity), increases photon collection efficiency (no pinhole, collection of full emission peak)^1,9,17^ and extends the depth of imaging because IR photons are more than 10-fold less scattered than visible light^9,18,19^. It should be noted that more than two photons can be absorbed at the same time which is exploited in three- and multi-photon microscopy providing an exciting avenue to deep tissue imaging^20–22^. Here, however, we focus on the more widespreadly used two-photon excitation.

TPE microscopy is not without its concerns and limitations. The high laser powers required might result in photodamage, yet the absorption of infra-red light by biological materials is considered low. It is worth noting that the efficiency of two-photon absorption (action cross section of the fluorophore in units of GM), a molecular property of the dyes, is small as compared to absorption in one photon but efforts to improve probes are constantly ongoing^14,23–25^. Given that the two-photon brightness is set by the absolute TPA cross-section and by the quantum yield, a fluorescent protein that is bright in one photon excitation might not appear as bright in TPE. Finally, the excitation spectra are not simply double the single photon excitation spectra; instead, the two-photon excitation spectra often show broadening, variable red-shifting and unexpected peaks due to different quantum mechanical selection rules governing one- versus two-photon excitation^26–28^. Two-photon absorption processes can often be non-intuitive, as the spectra and extinction coefficients are not simply related to the one-photon absorption properties of the dyes; Furthermore, computing two-photon absorption properties, especially for large molecules, is computationally difficult^29,30^. The spectra and absorption properties must be determined experimentally, as they may be highly dependent on the details of the experimental apparatus and the biological environment^26,28,31^.

A typical TPE setup is similar to a standard confocal laser scanning microscope without the confocal aperture (Fig. 1e)^9,32^. The light source (laser beam) is moved in the sample space using a galvanometric mirror (galvo) to raster scan each location in an optical section and construct an image, pixel-by-pixel, using the fluorescence collected onto a detector, typically a photomultiplier tube (PMT), an avalanche photodiode (APD), or a hybrid detector (e.g., GaAsP). Usually, the detector is moved just behind the objective as there is no need for a pinhole and rescanning the fluorescence in contrast to descanned one-photon confocal detection (Fig. 1e). This non-descanned detection method minimizes light losses by utilizing the entire light-sensitive area of the detector enabling the capture of scattered emission light and further minimizes light loss by decreasing the number of optical elements (mirrors, scan lens, tube lens, etc.) (Fig. 1d). In addition, fluorescence can be collected along the optical axis (from above in an inverted microscope configuration) allowing to collect fluorescence emitted in the direction of the excitation making use of signals otherwise not captured^33,34^.

**Fig. 2:**
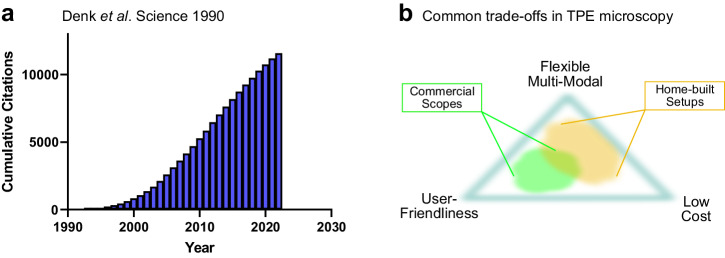
Popularity and needs of TPE microscopy. **a** Cumulative citations of reference^1^ Denk et al. Science (1990). This hallmark paper introduced TPE laser scanning microscopy and the use of non-linear microscopy to biological samples. Number of citations were exported from Google Scholar. **b** Needs and trade-offs in biological application of TPE microscopy: There is a demand for low-cost equipment that has to be balanced with user-friendliness (*ie*., turn-key instrumentation) and the flexibility (*e.g*., tuneable wavelengths, filters, photon counting applications). Commercial and home-built setups typically cover different regimes of the needs.

In the time since the first practical TPE microscope was demonstrated more than three decades ago, the demand for TPE in biological application never ceased (Fig. 2a) and new varieties for in vivo microscopy constantly evolve. The application of TPE microscopy has been empowered by the availability of robust, tunable, ultrafast IR lasers for excitation and by the availability of turn-key instrument solutions. At least 10 vendors offer TPE microscopes, with various specifications and custom options. Some instruments are specifically aimed at biologists driven by user-friendliness; whereas, others are motivated by users demanding more flexibility for customization, undaunted by the required expert knowledge in optics and hardware/software integration^35^. When choosing two-photon microscopy instrumentation, one needs to consider user-friendliness, costs, and flexibility (Fig. 2b). Furthermore, this choice should be driven by the biological phenomena to be investigated, and driven by a few questions:

Do I need access to the optical path?

Do I need the best possible single image, or do I need a large set of images (time series or z-stack)?

Do I need flexibility in wavelength or do I always use the same fluorophores?

Commercial vendors can help customize their solutions to the researcher’s needs but identifying these needs is paramount before deciding on equipment. Table 1 outlines a few of the available implementations with distinct features. Given these gateway offerings, it seems like a perfect time for optical veterans and for novice microscopists to start working with TPE microscopy.

**Table 1:** Non-exhaustive list of microscopy vendors offering TPE microscopy instrumentation highlighting unique features of each

Vendor	Model(s)	Notable features
3i	VIVO Multiphoton	- Very flexible, customisable platform - Integrated adaptive optics
Bruker	Ultima Series	- Rotating nosepiece - Remote focussing
Leica Microsystems	SP8 / Stellaris 8 DIVE	- Fully integrated with confocal LSM - Up to 4 non-descanned detectors - FLIM acquisition and analysis packages
Nikon	AX R MP	- Resonance scanner (720 fps at 512 × 16 pixels) - Tilting nosepiece - Array detector for increased SNR and resolution (NSPARC)
Olympus	FVMPE-RS	- Broad transmission 400 nm–1600 nm - Multichannel IR excitation
Prospective Instruments	MPX	- Compact and fully integrated system - FLIM capable
Sutter Instruments	MOM/DF Scope	- Moveable objective - Collection of emitted light above & below the sample for increased detection efficiency/SNR
Thorlabs	Bergamo II Series Mesoscope	- Flexible geometry with rotating body - Extended depth of field using Bessel beam - Dual plane imaging - Remote focussing - TPE random access modality
Zeiss	NLO module for LSM 980 (nonlinear optical microscopy)	- Integrated with LSM platform - Combination with airyscan offers increased resolution and speed

## Wider, faster, deeper - towards volumetric, intravital imaging with TPE microscopy

Complex biological processes occur across a wide range of time-scales, in all three spatial dimensions, which often limits comprehensive studies. Biological processes encompass phenomena like hormone release, calcium waves, differentiation, and apoptosis that unfold over time-scales ranging from milliseconds to hours or even days. While studying fixed samples at multiple time points is a potential approach to reconstruct such dynamics, the sheer number of samples required poses challenges for reproducibility. Consequently, current research necessitates imaging tools capable of rapidly capturing 3D samples at cellular resolution, enabling the investigation of dynamic, biological processes.

**Fig. 3:**
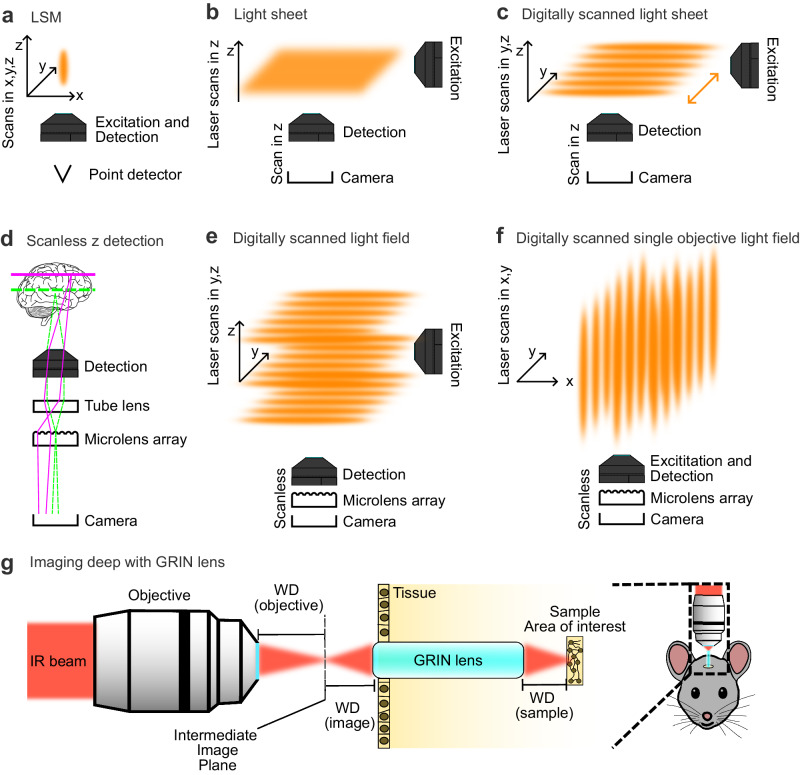
From laser point scanning to fast, wide and deep volumetric imaging in complex samples with TPE. **a** Scheme for conventional TPE laser scanning microscopy which requires scanning of the excitation/emission (orange ellipse) through x,y, and z dimension and collection of every single point onto a point detector. **b** Scheme for TPE light-sheet excitation and detection using a camera. Scanning is only required in z-dimension. **c** TPE scheme for digitally scanned light-sheet. The virtual sheet is created by scanning an extended TPE beam in y-dimension faster than the camera frame rate (orange double-arrow). Volumetric imaging is achieved by scanning in z-dimension. The camera detection integrates multiple scans of the beam in y-dimension into a single frame. **d** Scheme for scanless volumetric detection using light-field technology. The microlens array in front of the camera allows capturing z information from an excited volume at the expense of lateral resolution. A volume of illumination is generated by quickly scanning the virtual sheet along the z axis to excite and capture a volume in a single snapshot. “Human brain outline in lateral view” by an unknown author from Wikimedia Commons licensed under CC0 1.0. **e**, **f** Combination of light-field detection with two (**e**) or one (**f**) objective digitally scanned light-sheet excitation while selectively exciting a volume of interest. **g** TPE laser scanning microscopy in combination with an implanted GRIN lens for deep tissue imaging in live animals. WD Working distance, GRIN Gradient of refractive index.

Conventional TPE laser scanning microscopy, while offering excellent pixel resolution, suffers from slow imaging speed due to point-by-point scanning and the use of the same objective for excitation and detection (Fig. 3a). The most straight-forward solution towards increasing speed is scanning faster. Resonance galvanometric scanners are typically used for this purpose. They can achieve kHz scan rates, but are limited to specified field of views (FOVs) and sampling rates; they also shorten the pixel dwell time dramatically (*i.e*., reducing the time fluorescence can be recorded from each pixel). As the single-point scanning seems to be the main limitation several remedies have been developed to overcome this issue. Firstly, multi-focal schemes allow to distribute the focal area to different positions, for instance, through a micro lens array/disk, a beam splitter, beam shaping, or a set of mirrors^36–41^. An elegant scanless solution is to image an area instead of a single point in widefield-type illumination, for example, using temporal focussing^42,43^. To achieve sufficient photon density for TPE at the focal plane in widefield, the excitation pulses are first dispersed and then temporally compressed along the optical axis using a combination of low NA objective and reflective grating^44^. Such schemes allow simultaneous acquisition of multiple spatial locations or a plane at the cost of a lower signal-to-noise ratio (SNR) due to a smaller energy density at every focal volume. To spatially multiplex detection, multi-focal schemes employ cameras (CMOS, CCD sensors) as opposed to point detectors (PMTs, APDs) found in conventional laser scanning microscopes. This comes at the cost of decreased spatial resolution as scattered fluorescence may cause a blurred signal when detected on a camera. In summary, applying TPE to faster imaging modalities demands a balance between the photon density required for TPA while generating multiple focal points or an entire plane as in TPE light-sheet microscopy.

## Wider: TPE light-sheet fluorescence microscopy

Light-sheet fluorescence microscopy (LSFM) is an ideal approach to reliably capture transient biological processes of cells across hundreds of micrometers in 3D^45^. LSFM (also known as Selective Plane Illumination Microscopy) uses a thin sheet of light to excite fluorophores within a focal plane while reducing out-of-focus excitation. The excited focal plane is then captured as a 2D image using a CMOS or CCD sensor rather than a point detector, increasing both frame and volumetric acquisition rate. Because only the focal plane is excited, LSFM has true optical sectioning where background fluorescence is greatly reduced^46^. Optical sectioning of LSFM reduces photodamage by three orders of magnitude when comparing one-photon excitation LSFM to confocal microscopy^47^. When combined with TPE, photodamage, reduces by five-fold when compared with conventional TPE laser scanning microscopy^48,49^. TPE-LSFM further enhances imaging depth and SNR, while eliminating the need for visible excitation laser which can be a potential confounding factor in light-sensitive samples^50^ or behavioral experiments. Recent notable applications have applied TPE-LSFM to light-sensitive behavioral studies that require imaging large regions such as the whole brain during seizure^51^, sleep^52^, phototaxis^53^ and visual number sense^54^.

A challenge in TPE-LSFM is maintaining a sufficiently high photon density for excitation. In the first implementation, a light sheet is created by focusing the excitation beam through two cylindrical lenses to first create a line and then a sheet, fluorescence is detected orthogonally through a second objective on a camera^55^ (Fig. 3b). However, a cylindrical lens reduces the photon density, and consequently, decreases the fluorescence signal due to the quadratic dependence of TPE. To increase the photon density, the focal point of the TPE laser focus can be shaped (axially extended) into a micrometer-thin beam of light and can be quickly moved (scanned) laterally to create a “virtual light-sheet” that provides even illumination and higher photon density when compared to using cylindrical lenses^48,56–58^(Fig. 3c). The emitted fluorescence from the scanned area is integrated on the camera and the use of a low NA illumination objective partially mitigates the degradation of lateral resolution^48^.

Both LSFM modalities allow for increased spatial and temporal sampling (~4 orders of magnitude^51,59^) with the drawback of lower energy density and thus fluorescent light flux in a given focal point as compared to LSM approaches. Volumetric imaging is achieved by either mechanically moving the sample through the focal plane or simultaneously moving both the detection objective with a piezo element and excitation sheet using a galvanometric mirror to achieve volumetric acquisition speed of 0.5 Hz at 400 × 800 × 250 µm^3^ in 52 z-sections^60^. Higher volumetric acquisition speed can be achieved by replacing the piezo element with an electrotunable lens to gain speed of up to 5 Hz at 600 × 800 × 150 µm^3^ in 31 z-sections^51,61^.

## Faster: TPE light-field microscopy

Light-field microscopy (LFM) is an innovative technique that enhances the acquisition rate of 3D imaging by allowing targeted volume excitation without the need for time-consuming z-scanning. In traditional 3D imaging, the sequential scanning through voxels, lines, or planes creates a bottleneck, slowing down the imaging process. For example, LSFM requires scanning plane by plane (axially) to capture a 3D volume, thus this approach is limited by the exposure time required for each frame or z-section to gather sufficient photons. To address this issue, detection can be integrated with light-field technology. LFM captures both 2D spatial and 2D angular information of light emitted from the sample, effectively preserving 3D characteristics within a single camera frame^62^. LFM is typically implemented in widefield illumination with a microlens array (MLA) inserted in the image plane before the camera, redistributing the light on the chip based on the illuminated volume in the sample plane (Fig. 3d). While this technique sacrifices some lateral and axial resolution, a single snapshot can be subsequently reconstructed into a detailed 3D volume, effectively aligning the acquisition speed with the camera’s frame rate^63^.

Adding TPE to LFM increases the imaging depth while reducing out-of-focus illumination. Conventional implementations of LFM use widefield illumination^64^, however, in TPE a high photon density must be maintained. Therefore, different illuminations schemes similar to LSFM methods have been employed; for example, the excitation laser is extended axially and scanned laterally (Fig. 3e, f). The TPE beam scanning can be effectively applied in both multi-objective setups (Fig. 3e) and single-objective setups (Fig. 3f).

TPE LFM has the potential to non-invasively record millisecond events of thousands of cells across hundreds of cubic microns but the technology is still in its infancy. The volumetric imaging speed is constrained by the number of emitted photons from the fluorophores in the sample and detection efficiency of the camera. Brighter and faster fluorescent proteins and sensors are constantly being developed. The fastest calcium sensor can record single neuron firing speeds of up to 50Hz^65^. Similarly, higher quantum efficiency (>90%) cameras using backside illumination CMOS sensors are now more accessible (e.g., Sona-11 Series, Andor; ORCA-Fire, Hamamatsu). Another limitation of LFM is the lengthy volume reconstruction time which requires extensive computations and expert knowledge^64^. To speed up computation time, Guo et al.^66^ employed a Fourier imaging scheme to decrease reconstruction time by 100-fold. In conventional LFM, the MLA is placed at the native image plane (NIP)^63^. In Fourier LFM, the MLA is placed at the back focal plane of the Fourier lens. In the Fourier domain, the signals can be processed in parallel, meaning that multiple computations can happen simultaneously to decrease reconstruction time. There is a lot of ongoing work to improve lightfield technology to make it accessible and user-friendly, for example, a graphical user interface implementation for reconstruction is now available in napari^67^. We eagerly await to see the next generation of LFM technologies for imaging fast dynamic processes.

## Deeper: Periscopes from microlenses and GRIN lenses

While TPE microscopy improves penetration depth as compared to single photon excitation to hundreds of microns, optical aberrations in highly scattering tissue with non-uniform refractive index distribution degrade resolution. Incorporation of adaptive optics (AO) can help correct for these aberrations and allow for high-resolution imaging at depth^68–70^. However, many processes still remain out of reach. For example, to image millimeters deep into the cortex of an animal by conventional two-photon microscopy a considerable amount of scattering tissue must be surgically removed^71,72^. This invasive procedure provides access but might perturb the system. An emerging technology to provide higher imaging depths are gradients of refractive index (GRIN) lenses^73,74^. These optical elements are cylindrically shaped lenses varying the refractive index orthogonally to the optical axis. Implanting such a lens into tissue allows it to relay light from deep inside the sample to the imaging objective (Fig. 3g). Thus, it can be viewed as TPE endoscopy enabling us to investigate processes deep in tissue over weeks and months post-implantation of the GRIN lens. While getting the lens in place is an invasive procedure and the alignment of objective and GRIN lenses is challenging, it is thus far the only way to obtain fluorescence imaging information at such depths in intact non-transparent animals. The effective light throughput and resolution will be influenced by the properties of the GRIN lens such as numerical aperture or field curvature. Combination of this technology with AO^75^, improving the field of view^76^, imaging speed^77^ and resolution^78^ of GRIN lens systems and fiber based alternatives^79^ are active and exciting areas of research. Often, imaging requires immobilization of the specimen, for example the mouse, which can be circumvented by head-mounted TPE microscopes allowing to image brain activity in freely moving animals^80–82^.

## TPE microscopy and photon counting applications

**Fig. 4:**
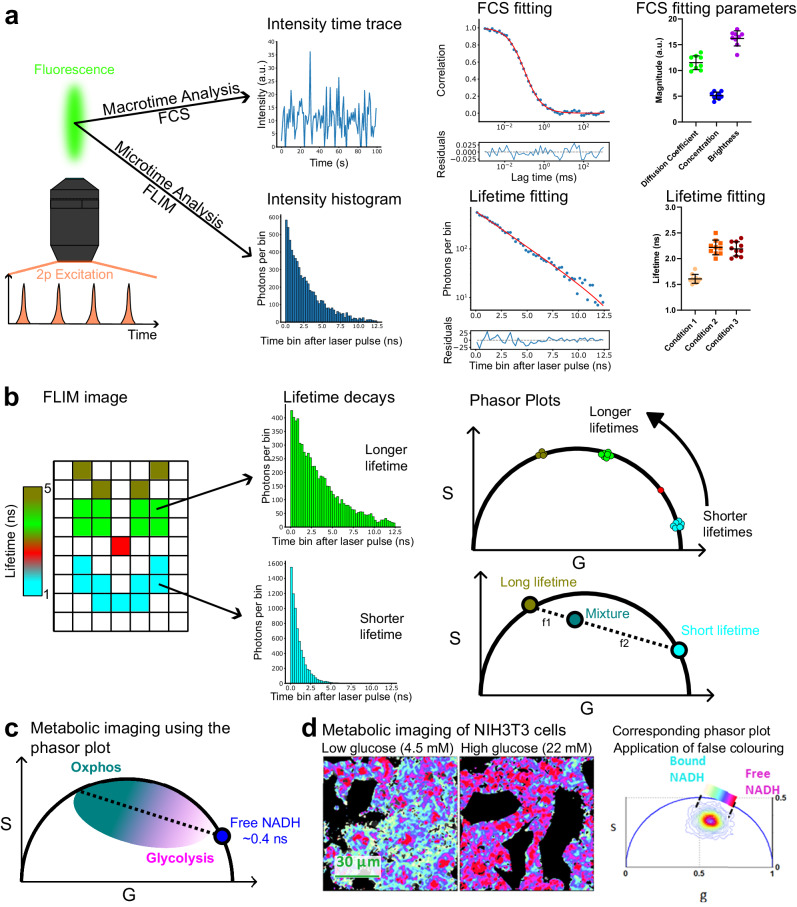
Combination of TPE with the photon counting techniques FCS and FLIM. **a** The pulsed excitation and resulting fluorescence in TPE microscopy can be combined with FCS and FLIM. In FCS the fluorescence time trace at a fixed point in space is analysed by means of temporal autocorrelation. Fitting the resulting autocorrelation curve by an appropriate model gives information on the diffusion coefficient, concentration and oligomeric state (brightness) of moving particles in the sample. Histogramming the photon arrival times after laser pulses allows for the investigation of the fluorescence lifetime of the observed fluorophores. **b** The pixels in FLIM imaging contain lifetime values in addition to the intensity values. For every pixel a lifetime decay (photon counting histogram can be calculated). A convenient way to compress the data is to map the decay curves via Fourier transform onto the phasor space. Mono-exponential lifetimes lie on the universal circle. Combination of lifetimes (or multi-exponential decays) map within the circle. **c** Application of Phasor-FLIM imaging to investigate metabolic state of cells by exploiting the autofluorescence of bound and unbound NADH. The more NADH is bound, the more oxidative phosphorylation (Oxphos) is performed revealing the metabolic phenotype (i.e., Oxphos versus glycolysis). Lifetimes can be false-colored using the phasor plot and remapped onto the FLIM image to identify spatial patterns. **d** Example of metabolic imaging of NIH3T3 cells in low or high glucose media (images on the left) and corresponding phasor coordinates of the pixel containing fluorescence (right). NADH was imaged using 740 nm excitation. False colouring using a magenta to blue look up table is applied to the phasor cloud remapping the pixels from phasor to image space. This allows the identification of pixels with more free NADH (meaning more glycolytic cells, short NADH lifetime) and pixels with more bound NADH (meaning more oxidative phosphorylation, long NADH lifetime). This panel was adapted from reference^117^ Stringari et al. (2012), PLOS ONE 10.1371/journal.pone.0048014 published under CC BY 4.0 https://creativecommons.org/licenses/by/4.0/.

Cells, tissues and organisms rely on the fast reorganization of biomolecules on time-scales beyond the capabilities of conventional imaging techniques. Fluorescence correlation spectroscopy (FCS) and fluorescence lifetime imaging microscopy (FLIM) offer access to these rapid time-scales as they are able to probe transient changes in molecular interactions or in the molecular environment. In FCS, photons are analyzed with respect to the start of the experiment (macrotime, seconds to minutes), and in FLIM, photon arrival times after exciting laser pulse (microtime, nano-seconds) are measured (Fig. 4a). Conveniently, these advanced photon counting techniques can be readily coupled to typical TPE laser scanning microscopes allowing us to eavesdrop on biological processes deep in tissue: FCS can be used to investigate diffusion dynamics, concentrations, or oligomerisation in vivo^11,83,84^, FLIM can be used to add extra contrast to the image and report on the local environment of the fluorophores^11,85^.

## TPE FCS

FCS is a point measurement technique to characterize molecular diffusion dynamics and concentrations. The laser focus is parked at a specified location (e.g. in the center of the field of view) and the fluorescence intensity over time is recorded, essentially performing a very fast imaging scan (sub-μs) of a single pixel (Fig. 4a)^83,86,87^. Analyzing the intensity fluctuations caused by molecules diffusing in and out of the focus yields information on kinetic (diffusion coefficient) and thermodynamic (concentration, brightness/oligomeric state) properties of the system. These parameters are obtained by fitting the autocorrelation curves to an appropriate model^83,87,88^ and can then be statistically compared for different conditions.

TPE naturally extends the capabilities of FCS because it (i) inherently constraints the fluorescence to a sharp sub-femto-liter volume, necessary to obtain the intensity fluctuations to analyze for FCS (ii) results in no out-of-focus excitation, causing less photodamage, less accumulative photobleaching (less change in concentration over time), and less background fluorescence, (iii) shows no artifacts from scattered laser light as excitation and emission wavelength are far apart, and (iv) improves penetration through thicker samples^89,90^. TPE FCS has been used to study diffusion dynamics in the cytoplasms or membranes of living cells^89,91–93^, embryos^94^, and throughout organisms^95,96^. We expect with the availability of turn-key instruments to see a renaissance of the application of this technology as well as the increase in use of related techniques such as scanning FCS (sFCS)^87,90,97,98^ or raster image correlation spectroscopy (RICS)^99,100^ that provide spatial context to the FCS data.

## TPE FLIM

FLIM reports intensity and fluorescence lifetime for every pixel providing additional means to generate contrast in the image. The fluorescence lifetime refers to the time a fluorophore spends in an excited state S_1_ before returning to the ground state S_0_ (see also Fig. 1b). The fluorescence lifetime is a molecular property of the fluorophore and its local environment^101,102^. It can be used to add contrast to the image, to allow discrimination of two dyes with similar emission wavelengths but different lifetimes (for example removal of autofluorescence with characteristic lifetime), or to report on changes in the local environment of the fluorophore such as viscosity, pH, or binding^102^ (Fig. 4b).

FLIM data can be recorded in the time domain (using a pulsed laser and photon counting) or in the frequency domain (using excitation modulation and measuring the phase shift between excitation and emission). Both approaches can be coupled to TPE laser scanning microscopy in a readily integrated microscope or added as an LSM upgrade kit (typical vendors for these options include: Becker and Hickl, PicoQuant, ISS, Leica Microsystems). In the more widely used time domain measurements, the time delay of emitted photons with respect to the excitation pulse is analyzed for every pixel (microtime, Fig. 4a)^11,103,104^. Building a histogram of the emitted photon arrival times after laser pulse is called time correlated single photon counting (TCSPC). The decay curve is fitted with appropriate models (e.g., multi-exponential function) to obtain the fluorescence lifetime per pixel (Fig. 4a bottom, Fig. 4b left). Obtaining valid results from this approach requires careful attention to the photon statistics; collecting a sufficient number of photons per pixel to yield an accurate fluorescence lifetime estimate is crucial and can be time consuming. Further, it is important to consider that more photons are required to differentiate small lifetime differences (e.g., 1.2 ns versus 1.4 ns) as compared to large lifetime differences (e.g., 1 ns and 5 ns)^85,105^. Therefore, FLIM acquisitions are usually an order of magnitude slower, as compared to using the intensity image (i.e., in conventional confocal imaging), hampering studies of fast (sub-second) biological processes with FLIM. However new photon counting hardware (commercial options include: Becker and Hickl, SPC-QC-008; Leica Microsystems, FALCON; PicoQuant, PicoHarp 330) and corrections for photon counting at high photon fluxes (deadtime and pile-up corrections)^106,107^ are becoming available.

Analyzing FLIM data can alternatively be performed using the fitting-free phasor approach (Fig. 4b, right)^108^. In essence, the phasor analysis uses a Fourier transform to approximate the fluorescence decay in each pixel. In this process a decay curve (often > 200 photon bins per pixel) is compressed to two phasor coefficients (real and imaginary part of the phasor, usually termed G and S respectively, see Fig. 4b)^109^. This process is performed for every pixel. Filtering the phasor coefficients (the G and S images, *e.g*., using a median filter) helps improve the SNR^108,110^. Mono-exponential lifetimes fall on the semi-circle whereas bi-exponential lifetimes fall within. A combination of two mono-exponential lifetimes maps within the circle but can be decomposed into the original components as well as their fractions estimated (Fig. 4b bottom). The key point of the phasor transform is that pixels showing similar lifetimes / fluorescence properties will have similar G and S values on the phasor plot and can be analyzed together. These pixels can be far apart in the original image. In this way the phasor transform helps to elucidate spatial patterns, number of components with distinct lifetimes, and their interactions in the sample. No fitting of the data is performed, making it a fast, unbiased and convenient way to explore the data rather than focusing on determination of exact lifetimes.

Imaging of autofluorescent, endogenous compounds can provide insights into cellular physiology. However, these molecules often need to be excited in the UV range (using one photon excitation). TPE FLIM enables such measurements without exposing the sample to extended UV irradiation. While TPE FLIM has also been used with fluorescent biosensors^111–114^, we focus here on the application in label-free microscopy using intrinsic biomarkers^115,116^. In metabolic imaging, for example, the cofactors NADH and NADPH are excited around 740 nm^117–120^. Their lifetime can be used to infer metabolic state of a cell or tissue (Fig. 4c)^85,117,121,122^ as these compounds change fluorescence lifetime when binding to metabolic enzymes of the oxidative phosphorylation pathway (Oxphos)^123,124^. The more NADH is free in a cell, the less Oxphos is in progress, the more glycolysis is performed (Fig. 4c). Using FLIM, cells or tissues can be profiled for metabolic state under various conditions such as during glucose shock (see example in Fig. 4d)^117^, infection^125,126^, differentiation^127–130^, diabetes^131^, drug treatments^117,132^, or in the context of neuro-pathophysiology^133–137^. While examining NADH lifetime can provide valuable insights, imaging conditions especially when fixation or embedding is required need to be carefully evaluated^138^. As the autofluorescence of this endogenous compound is dim, the phasor analysis has over the past years evolved to the gold standard to process and analyze such low SNR FLIM data (Fig. 4b, c)^108,139^

## Maximizing SNR within the limitations of TPE

Equally important to enhancing temporal and spatial sampling is the pursuit of optimal image quality, SNR, while ensuring the health and integrity of the sample. These factors need to be balanced to ensure collection of meaningful biological data. This section addresses two frequently underestimated yet resolvable concerns: photodamage and photo selectivity.

### Optimizing SNR and photodamage

Understanding the processes involved in the photodamage from TPE can be challenging as it introduces photodamage both linearly and nonlinearly with increasing excitation power. Generally, photodamage results from two distinct processes: photothermal and photochemical effects^140^. Photothermal damage results from laser heating outpacing the dissipation of the heat, and typically follows a time-averaged photon absorption process (linear effect)^141–144^. Photochemical effects result from the ionization of molecules and formation of reactive oxygen species (ROS) through multiphoton absorption processes (nonlinear effect)^140,145–152^.

One approach to minimize nonlinear photodamage is to decrease the photon density of a single pulse (or peak power). The peak power $({P}_{{peak}})$ can be described by:

1 $${P}_{{peak}}\propto \frac{{P}_{{mean}}}{\tau \cdot f\,}$$

Where laser pulse repetition rate = $f$, mean laser power (pulse + inter-pulse interval) = ${P}_{{mean}}$, and pulse width = $\tau$.

The easiest means to decrease linear photodamage is to decrease ${P}_{{mean}}$, ideally while keeping ${P}_{{peak}}$ constant, as fluorescence signal $(S)$ is dependent on both ${P}_{{peak}}$ and ${P}_{{mean}}$^153–155^:

2 $$S\propto \frac{{{P}_{{mean}}}^{2}}{\tau \cdot {f}^{2}}\,$$

To maintain or increase signal while minimizing damage, adjusting ${P}_{{peak}}$ or ${P}_{{mean}}$ by modifying only the laser output at the source is inadequate. A far better approach is to utilize a pulse compensator to change pulse width ($\tau$) to increase ${P}_{{peak}}$ without increasing ${P}_{{mean}}$, and/or a pulse picker to decrease the repetition rate $f$ to maintain ${P}_{{peak}}$ but decrease ${P}_{{mean}}$^154^.

Adjustments of ${P}_{{peak}}$ and ${P}_{{mean}}$ must be empirically determined for different experiments because the TPE focal point (photon density) at the sample may vary. The variation is due to the sample type, objective NA, and illumination scheme (point scanning, lightsheet, lightfield, temporal focusing)^51,143,153,156^. In practice, we adjust ${P}_{{peak}}$ and ${P}_{{mean}}$ by first determining what type of photodamage is occurring within the sample. Linear photodamage from TPE is similar to photoablation, where the damaged area results in a small cavity, typically appearing as a dark non-fluorescent region^157^. Nonlinear photodamage is more complex to judge and may result in either darkening or intensifying the local signals due to the photochemical effects. Photobleaching is only one of the signatures of photochemical damage, and the most accessible sign of nonlinear photodamage^158,159^. However, the absence of photobleaching does not necessarily guarantee the absence of nonlinear photodamage as other molecules may be affected before the fluorophore^160^. Another challenge in determining nonlinear photodamage, are studies using Ca^2+^ indicators, as photobleaching can be difficult to assess because the baseline fluorescent change can be a complex mixture of linear and nonlinear effects^143,144^. One convenient method to determine the photodamage type, is to image the sample with a continuous wave laser of the same ${P}_{{mean}}$ (e.g., by disabling mode-locking of the laser source). It is good practice after TPE microscopy to perform a viability or behavioral assay to determine the health of the sample^160^.

### Selective excitation by polarized light

**Fig. 5:**
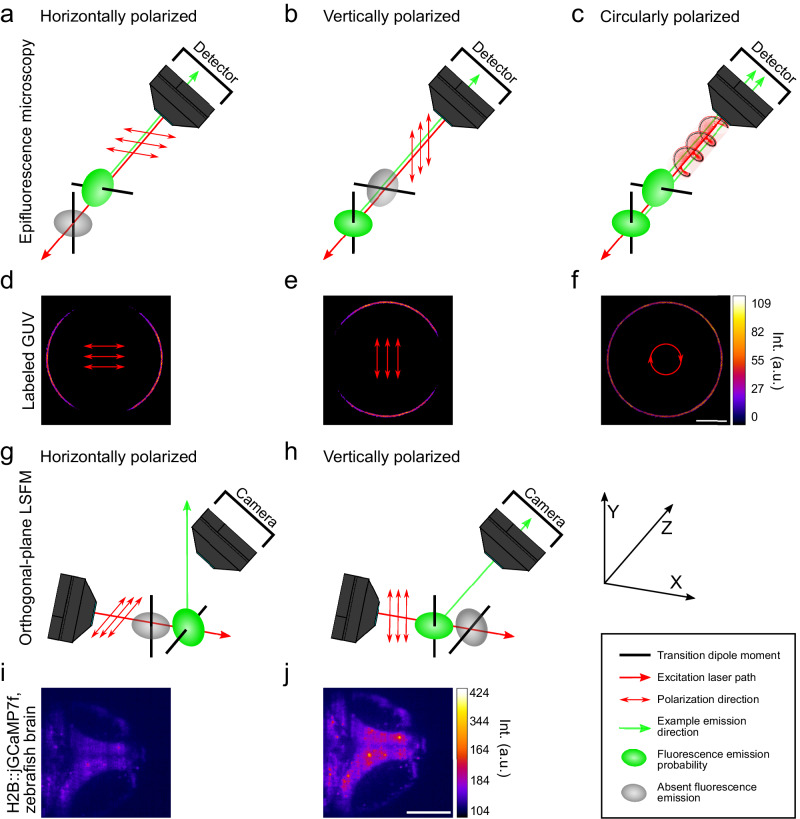
Optimization of excitation laser polarization for single and multi-focal microscopy. **a**–**c** Epifluorescence microscopy. Excitation and detection light travel through the same objective. Horizontally polarized excitation laser (**a**) vertically polarized excitation laser (**b**) circularly polarized excitation laser (**c**). **d**–**f** Example image of equatorial plane of a giant unilamellar vesicle (GUV) taken with epifluorescence microscopy. GUV consists of an unsaturated phospholipid (1,2-dioleoyl-sn-glycero-3-phosphocholine, DOPC), is labeled with Fast-DiO and excited with 950 nm. Horizontally polarized (**d**) vertically polarized (**e**) circularly polarized, simulated image (**f**). Scale bar, 10 µm. **g**, **h** Multifocal orthogonal LSFM. Excitation and detection light travel through different objectives. Horizontally polarized excitation laser (**g**) vertically polarized excitation laser (**h**). **i**, **j** Example image of a 7-day post-fertilization zebrafish midbrain expressing pan-neuronal H2B::jGCaMP7f^194^ taken with orthogonal LSFM. The sample is illuminated by a single lightsheet from bottom of the image at 920 nm with 175 mW of power. Example image is averaged across 30 s showing a single z-plane of a volumetric time series taken at 1 volume per second. Scale bar, 100 µm. **a**–**c**, **g**, **h** Green arrows show one possible radial emission direction; Green ellipsoid has the highest fluorescence emission probability perpendicular to the dipole moment, at the ellipsoid’s equator. Circularly polarized light was adapted from “Clockwise circularly polarized light” by Dave3457, Wikimedia Commons, licensed under CC BY-SA 4.0.

An often-overlooked phenomenon in fluorescence microscopy is photoselection caused by the linear polarization of the excitation light. This phenomenon can preferentially excite certain fluorophore orientations, due to their dipole moments, leading to fluorescence emission anisotropy^11^. The orientation of the transition dipole moment of the fluorophore not only describes the shift in electron density upon excitation but also determines dye excitation efficiency^161^. The most efficient excitation takes place when the transition dipole moment of the fluorophore aligns with the polarization direction of the light. Fluorescence emission is also oriented, with the photons emitted in the plane perpendicular to the transition dipole moment (Fig. 5a, b). For such fluorescence emission anisotropy to occur, the fluorescence lifetime must be shorter than the rotational diffusion time (the average time required for the molecule to complete a rotation). The rapid tumbling motions of dyes in solution would randomize these orientations, which is why the contribution from photo-selection is often under-appreciated.

Because the laser light typically used to excite fluorophores is linearly polarized, the preferential excitation of fluorophores that are bound to or embedded in targets result in intensity variations in the sample. In epifluorescence microscopy, this can be easily demonstrated with a labeled model membrane system, such as giant unilamellar vesicles (GUVs). A fluorophore, such as Fast-DiO, oriented radially within the GUV membrane shows the expected fluorescence emission anisotropy (Fig. 5d, e); the non-selected direction is almost completely dark. A quarter-wave plate offers an easy solution for this issue in epifluorescence microscopy by creating circularly polarized light^162,163^ (Fig. 5c, f), permitting the laser to excite all dye orientations.

Line-scanning microscopy techniques like orthogonal-plane LSFM or oblique plane microscopy (OPM) can leverage photoselection to minimize laser power and photodamage while maximizing the fluorescence signal intensity. Unlike in epifluorescence, the solution is not to excite as many fluorophores as possible but to excite only those fluorophores that most efficiently emit fluorescence light towards the camera direction^164^. For example, one can selectively excite fluorophores that will emit fluorescence light radially on a 2D plane by using a half-wave plate to control the directions of linearly polarized light. If not optimized in this fashion, the detected fluorescence can be reduced for the same number of excited dyes, because of their decreased emission towards the detection camera (Fig. 5g). Optimal efficiency of fluorescence collection should result from linearly polarized light oriented to best excite fluorophores that will emit fluorescence light radially on the xz plane (Fig. 5h). This effect has been demonstrated using an orthogonal-plane LSFM to image jGCaMP7f calcium indicators in a 7-day post-fertilization zebrafish midbrain (Fig. 5i, j). While its impact in single objective TPE OPM has yet to be demonstrated, we expect it should be similarly improved as compared to two objective orthogonal LSFM^165,166^. In short, optimizing the linear polarization direction during one- or two-photon excitation will maximize the fluorescence signal collection, which is particularly important for TPE because the higher laser powers increase the risk of photodamage.

### Image quality metric

The imaging community has strived to achieve FAIR (Findability, Accessibility, Interoperability, and Reusability) practices. The impact of FAIR practices has been demonstrated impressively in structural biology, where every structure deposited into the protein database is accompanied by quality metrics such as resolution, R-value etc. This allows for meta-analyses, joint refinement of structures, fostering collaborations, and direct comparisons between datasets. Similar efforts in fluorescence microscopy have been underway, but with far less acceptance in the field^167,168^. Likely, it will take more proactive involvement of publishers and funding agencies to mandate FAIR practices and to convince the user community. We believe that this would represent a game changer for reproducibility and transparency, making us hope it will not be too long before the TPE microscopy community adapts standardization routines, published along the data, that will allow us to quantitatively compare datasets and results.

## Quo vadis? What’s next?

Application, further advancement, and replacement of technologies are best dictated by the biology under investigation. For TPE microscopy such improvements have largely been driven by the neuro-science community and their endeavors to map whole-brain activity. This has resulted in technology to go faster and deeper, as well as pushed more towards volumetric imaging.

Instrumentation including optics and electronics for TPE microscopy as well as the acquisition schemes are constantly improving. This has been carried by an open-source culture that helps drive innovation and affordability of custom setups^169,170^. Technology advancements such as the use of single photon avalanche diode (SPAD) arrays (instead of single point detectors) should provide a wealth of new information in laser scanning microscopes^171,172^. While technology improvements will keep pushing the edge of possibilities a few milliseconds and microns at a time, in our opinion, ground-breaking, biological insights will arise from:

i. Applying appropriate technologies to the right biological question/system
ii. New probes, labeling strategies, and optogenetic modifications
iii. Better image and signal processing algorithms readily integrated in TPE microscopes

The choice of microscopy modality determines the dynamic range and information content that can be extracted from biological experiments. Imaging with TPE has many advantages over single-photon excitation and has become the preferred method for deep tissue imaging. Furthermore, it can be straight-forwardly combined with harmonic generation microscopy allowing for additional label-free contrast in tissues^115,173^. Now the challenge is to decide in which applications the advantages outweigh the expenses of TPE and which modalites (e.g., point scanning versus light-sheet) should be considered. Not every sample necessitates the most advanced imaging methodology, as the same conclusions might be reached with a simpler approach. We would like to emphasize that designing the experiment and choosing the required imaging modality carefully is crucial to discovery and can save a lot of time and money. Typical questions to keep in mind should include:

What spatial and temporal resolution do I need?

What field of view and imaging depth do I need?

Does cross-excitation matter?

Do I need optical sectioning?

What would the disadvantages in one-photon excitation be?

Developments of new probes, sensors, and labeling strategies will enable new insights into biological processes. Currently, brighter, more photo-stable fluorescent proteins and sensors (*e.g*., calcium or voltage imaging) already revolutionize what can be measured on conventional instruments^174–179^. An exciting direction is the use of red-shifted probes to exploit the red part of the visible light spectrum (>600 nm)^180,181^. Red-shifted probes allow for less scattering and absorption as well as better penetration depth. While development of brighter red fluorescent proteins is well underway and new detector technology starts to overcome the low quantum efficiency of standard PMTs in this regime, further improvements will have a major impact and essentially add another channel to experiments^174,175,179^. Moreover, the use of smart probes and biosensors that report on changes in sample properties such as pH value, temperature or viscosity will provide new insights on the local environment around a protein of interest^182–184^. Similar to metabolic imaging, using the right probe can unlock more information than just the spatial distribution of the fluorophore. Furthermore, photostimulation (*e.g*., uncaging of neurotransmitters or calcium, optogenetic manipulation through channelrhodopsins) offers exciting strategies to precisely control cellular signaling in space and time^185–188^.

TPE spectra are broad and a single wavelength can excite multiple fluorophores (cross excitation). The emission spectra of the excited fluorescent proteins can be highly overlapping which is a challenge in any multi-color fluorescence microscopy experiment. Recent advances in unmixing algorithms promise to overcome this issue and make most of overlapping emission signals^189–191^. Hyper-spectral approaches use, for example, detector banks or prisms and cameras to record emission intensity in spectral bins instead of a single channel. This allows for unmixing of different fluorophores and removal of autofluorescence^192,193^. Furthermore, the combination of spectral and lifetime detection offers new avenues to multiplexing in biological imaging^139,189^. We believe that these technologies will be integrated in turn-key systems and improve TPE imaging. However, we do emphasize that code and analysis pipelines should be open-source and easily accessible to anyone; this is crucial for quality control and reproducibility.

Overall, it is the perfect time to dive deeper into biological tissues using TPE microscopy. We hope to leave the reader with some two-photon excitement for recent advancements in technology, their applications, and an appreciation for their current limitations.
